# Predicting Empathy From Resting State Brain Connectivity: A Multivariate Approach

**DOI:** 10.3389/fnint.2020.00003

**Published:** 2020-02-14

**Authors:** Leonardo Christov-Moore, Nicco Reggente, Pamela K. Douglas, Jamie D. Feusner, Marco Iacoboni

**Affiliations:** ^1^Ahmanson-Lovelace Brain Mapping Center, University of California, Los Angeles, Los Angeles, CA, United States; ^2^Brain Research Institute, University of California, Los Angeles, Los Angeles, CA, United States; ^3^Department of Psychiatry and Biobehavioral Sciences, Jane and Terry Semel Institute for Neuroscience and Human Behavior, University of California, Los Angeles, Los Angeles, CA, United States; ^4^Institute for Simulation and Training, University of Central Florida, Orlando, FL, United States; ^5^Brain and Creativity Institute, School of International Relations, University of Southern California, Los Angeles, CA, United States; ^6^The Tiny Blue Dot Foundation, Santa Monica, CA, United States

**Keywords:** empathy, empathic concern, fMRI, resting state, connectivity, machine learning, experience sharing, mirroring

## Abstract

Recent task fMRI studies suggest that individual differences in trait empathy and empathic concern are mediated by patterns of connectivity between self-other resonance and top-down control networks that are stable across task demands. An untested implication of this hypothesis is that these stable patterns of connectivity should be visible even in the absence of empathy tasks. Using machine learning, we demonstrate that patterns of *resting state fMRI connectivity* (i.e. the degree of synchronous BOLD activity across multiple cortical areas in the absence of explicit task demands) of resonance and control networks predict trait empathic concern (*n* = 58). Empathic concern was also predicted by connectivity patterns within the somatomotor network. These findings further support the role of resonance-control network interactions and of somatomotor function in our vicariously driven concern for others. Furthermore, a practical implication of these results is that it is possible to assess empathic predispositions in individuals without needing to perform conventional empathy assessments.

## Introduction

Empathy is a complex phenomenon that allows us to share in (or *resonate* with) the internal states of others, as well as *infer* their beliefs and intentions (Decety and Jackson, 2006; Zaki and Ochsner, 2012; Christov-Moore and Iacoboni, 2016). It has been suggested that empathy’s *purpose*, in both humans and non-human animals, can be broadly divided into two categories: First, promoting pro-social, cooperative behavior via empathic concern for others and second, inferring and predicting the internal states, behavior and intentions of others (Davis, 1983; Preston and De Waal, 2002; Smith, 2006). In this study, we will focus primarily on elucidating the mechanisms underlying empathic concern.

In order to fulfill these purposes, empathy relies in part on our brains’ ability to reflexively process the observed or inferred experiences of others much in the same way we do our own, causing us to respond vicariously to their pain, visceral sensations, and emotions, and simulate their behavior within our own motor systems (reviewed in Zaki and Ochsner, 2012). Furthermore, this phenomenon extends beyond perception to behavior: we tend to reflexively mimic each other’s behavior, often without our knowledge (Chartrand and Bargh, 1999; Lakin and Chartrand, 2003; Sperduti et al., 2014), a process that can occur involuntarily when certain prefrontal control areas are damaged (Lhermitte, 1983; De Renzi et al., 1996). We will refer to this reflexive and embodied ability to simulate others as “self-other resonance” (Eisenberg and Fabes, 1990; Batson, 1991; Christov-Moore and Iacoboni, 2016), or *resonance* for short. The most likely neural substrate for resonance appears to be “neural resonance” (Zaki and Ochsner, 2012), the phenomenon of shared neural representations for the perception and experience of disgust (Wicker et al., 2003; Jabbi et al., 2007), somatosensation (Singer et al., 2006; Bufalari et al., 2007; Masten et al., 2011), emotion (Carr et al., 2003; Pfeifer et al., 2012), and motor behavior (Keysers and Fadiga, 2008; Iacoboni, 2009). Not surprisingly, neural resonance has been repeatedly associated with self-reported measures of trait empathy (Jabbi et al., 2007; Avenanti et al., 2009; Pfeifer et al., 2012) and is predictive of pro-social behavior (non-strategic generosity in economic games: Christov-Moore and Iacoboni, 2016; harm aversion in moral dilemmas: Christov-Moore et al., 2017b; donations to reduce pain in another: Gallo et al., 2018; helping behavior: Hein et al., 2011; Masten et al., 2011; charitable donations: Ma et al., 2011), suggesting that our resonance with others may underlie our *empathic concern* (and hence prosocial inclinations) for others.

In further support of a common substrate, prosocial inclinations and self-other resonance are similarly modulated by others’ closeness, status, group affiliation, and perceived trustworthiness (Chartrand and Bargh, 1999; Lakin and Chartrand, 2003; Singer et al., 2006; Gu and Han, 2007; Lamm et al., 2007; Hein and Singer, 2008; Loggia et al., 2008; Cheng et al., 2010; Guo et al., 2012; Reynolds-Losin et al., 2012, 2014, 2015; Sperduti et al., 2014; Schmälzle et al., 2017). This is likely due to top-down control processes that integrate contextual information and conscious appraisal with affective, somatosensory and motor processes into behavior and decision-making, implemented by prefrontal and temporal systems including the temporoparietal junction (TPJ) as well as dorsomedial and dorsolateral prefrontal cortex (DMPFC and DLPFC) (Miller and Cohen, 2001; Banks et al., 2007; Decety and Lamm, 2007; Cho and Strafella, 2009; Spengler et al., 2010; Brighina et al., 2011; Volman et al., 2011; Tassy et al., 2012; Winecoff et al., 2013). Not surprisingly, these control systems overlap considerably with those associated with conscious appraisal processes and inferential forms of empathy or mentalizing (Mahy et al., 2014). The nature of this control seems to be inhibitory: a recent study has found that disruptive neuromodulation of DMPFC and DLPFC caused a decrease in the inhibitory influence of context on prosocial behavior (Christov-Moore et al., 2017a). Evidence suggests that this top-down control of resonance is also continuously engaged: lesions to prefrontal cortex are associated with compulsive imitative behavior, suggesting that, for normal behavior to exist, some mechanisms to control resonance are always at play, unless damaged (Lhermitte, 1983; De Renzi et al., 1996). Within the context of empathy, resonance and control may exist most often as clusters within a single integrated system.

Indeed, the neural bases of resonance and control processes are not cleanly separable within cognitive function. Recent research suggests that somatomotor and affective processing contribute to our evaluations of others’ internal states, beliefs, and intentions (Gallese, 2007; Schulte-Rüther et al., 2007; Frith and Singer, 2008; Obhi, 2012; Christov-Moore and Iacoboni, 2016; Christov-Moore et al., 2017a), as well as our decisions about others’ welfare (Greene, 2001; Camerer, 2003; Van’t Wout et al., 2006; Oullier and Basso, 2010; Hewig et al., 2011; Christov-Moore et al., 2017a, b). Conversely, top-down control processes are increasingly implicated in the contextual modulation of neural resonance (Singer et al., 2006; Gu and Han, 2007; Lamm et al., 2007; Hein and Singer, 2008; Loggia et al., 2008; Cheng et al., 2010; Guo et al., 2012; Reynolds-Losin et al., 2012, 2014, 2015). Many studies have reported concurrent activation of and connectivity between ROI’s within one or more cortical networks associated with resonance and top-down control, such as during passive observation of emotions or pain (Christov-Moore and Iacoboni, 2016), passive observation of films depicting personal loss (Raz et al., 2014), reciprocal imitation (Sperduti et al., 2014), tests of empathic accuracy (Zaki et al., 2009), and comprehension of others’ emotions (Spunt and Lieberman, 2013). Co-existence of bottom-up resonance and top-down control mechanisms can be documented even at the level of TMS-induced motor evoked potentials (MEPs), a functional readout of motor excitability (Gordon et al., 2018). Thus, the neural instantiation of resonance and control may rely on systems that operate like connected clusters in a network, with different modes and configurations of function (Fox and Friston, 2012).

On the basis of this evidence, we propose that individual differences in empathic function (*particularly empathic concern for others*) arise in large part from stable, characteristic interactions between resonance and control processes at the neural level (Christov-Moore and Iacoboni, 2016; Christov-Moore et al., 2017a). This view is in line with studies showing that individual differences in active, task-relevant network configuration are reflected in intrinsic functional connectivity patterns (Smith et al., 2009; Tavor et al., 2016). We propose that in adults, these individual differences in empathic function should be apparent in resting connectivity (i.e. in the absence of empathy-evoking stimuli), much in the way a river carves out a characteristic pattern in bedrock over time. If so, this could have implications for understanding differences in empathic functioning without needing to probe participants with specialized tasks or questionnaires. Thus, we approached this current work with specific and general hypotheses: Specifically, we hypothesized, in line with our prior studies on the neural bases of prosociality, that patterns of functional connectivity between resonance and top-down control networks (proposed in Christov-Moore and Iacoboni, 2016) would predict participants’ empathic concern for others. In contrast to the previous univariate analyses, our goal was to derive a multivariate understanding of how empathy is represented by connectivity in these networks. Following work on resting-state and empathy (e.g. Cox et al., 2012), in a more exploratory fashion, we hypothesized that resting connectivity patterns within and between other cortical networks could also predict levels of trait empathy, with particular attention to the somatomotor network, which has been linked to many forms of prosociality (non-strategic generosity in economic games: Christov-Moore and Iacoboni, 2016; harm aversion in moral dilemmas: Christov-Moore et al., 2017b; donations to reduce pain in another: Gallo et al., 2018; helping behavior: Hein et al., 2011; Masten et al., 2011; charitable donations: Ma et al., 2011).

Additionally, there is a great deal of evidence for sex differences in empathy across a broad array of measures and associated brain function (reviewed in Hoffman, 1977; Eisenberg and Lennon, 1993; Christov-Moore et al., 2014; although, for negative/null results, see Lamm et al., 2011). For example, females display greater concern and sympathetic behavior toward others in real and hypothetical scenarios (Eisenberg and Lennon, 1993; Mesch et al., 2011; Christov-Moore et al., 2014; Friesdorf et al., 2015). Females also show greater vicarious somatosensory responses to the sight or knowledge of another person in pain or distress (Singer et al., 2006; Yang et al., 2009; Groen et al., 2013; Christov-Moore et al., 2014; Christov-Moore and Iacoboni, 2019), and exhibit greater facial mimicry when viewing emotional facial expressions (Sonnby-Borgström, 2002). For this reason, we controlled for sex within the primary analysis predicting trait empathy.

Taken together, previous studies suggest that resonance and control processes’ interactions, as measured via connectivity, may be the basis for individual differences in empathic concern for others, and that these interactions are relatively stable across task demands, sufficiently so that they should be observeable at rest. Thus, we sought to test two families of hypotheses: (I) our primary, theory-driven hypothesis that Resonance and Control interconnectivity at rest predicts trait Empathic Concern, and (II) our exploratory, theory-consistent but broader hypothesis that trait empathy can be predicted from resting intra- and inter-connectivity of intrinsic brain networks.

## Materials and Methods

### Participants

Participants were 58 ethnically diverse adults aged 18–35 (30 female, 28 males) recruited from the local community through fliers. All recruitment and experimental procedures were performed under approval of University of California, Los Angeles (UCLA)’s Institutional Review Board, in accordance with the ethical standards of the institutional and/or national research committee and with the 1964 Helsinki declaration and its later amendments or comparable ethical standards. Informed consent was obtained from all individual participants included in the study.

Eligibility criteria for participants included: right handed, no prior or concurrent diagnosis of any neurological, psychiatric, or developmental disorders, and no history of drug or alcohol abuse. These were all assessed during preliminary screening interviews conducted by phone at the time of recruitment.

### Trait Empathy Assessment

Participants filled out the *Interpersonal Reactivity Index* (IRI) at the end of each experimental session in a closed room, unobserved. The IRI (Davis, 1983) is a widely used (Avenanti et al., 2009; Pfeifer et al., 2012) and validated (Litvack-Miller et al., 1997) questionnaire designed to measure both “cognitive” and “emotional” components of empathy. It consists of 24 statements that the participant rates on a five-point scale ranging from 0 (Does not describe me very well) to 5 (Describes me very well). The statements are calculated to test four theorized subdimensions of empathy:

*Fantasizing Scale (FS)*: the tendency to take the perspective of fictional characters.
*Empathic Concern (EC)*: sympathetic reactions to the distress of others.
*Perspective Taking (PT)*: the tendency to take other’s perspective.
*Personal Distress (PD)*: aversive reactions to the distress of others.

Participants’ scores were summed for each sub-dimension (measured by six items) to make four scores per participant. Cronbach’s alpha, a measure of reliability, was assessed for the IRI using SPSS (FS = 0.752, EC = 0.792, PT = 0.816, PD = 0.839) (Ibm Corp, 2017).

### Functional MRI Data Collection

All neuroimaging data were acquired via a series of MRI scans conducted in a Siemens Trio 3T scanner housed in the Ahmanson-Lovelace Brain Mapping Center at UCLA. Resting data were collected while participants passively observed a white fixation cross on a black screen. They were instructed only to “Look at the fixation cross and just let your mind wander.” Resting-state functional images were acquired over 36 axial slices covering the whole cerebral volume using an echo planar T2^∗^-weighted gradient echo sequence (6 min; TR = 2500 ms; TE = 25 ms; flip angle = 90°; matrix size = 64 × 64; FOV 20 cm; in-plane resolution = 3 mm × 3 mm; slice thickness = 3 mm/1 mm gap). A T1-weighted volume was also acquired in each participant (TR = 2300 ms, TE = 25 ms, TI = 100 ms, flip angle = 8°, matrix size = 192 × 192, FOV = 256 cm, 160 slices, voxel size 1.3 × 1.3 × 1.0 mm).

### Functional MRI Preprocessing

Functional MRI preprocessing was performed in FEAT (FMRI Expert Analysis Tool), part of FSL (FMRIB’s Software Library^1^). After motion correction using MCFLIRT, images were temporally high-pass filtered with a cutoff period of 100 s (equivalent to 0.01 Hz) and smoothed using a 6 mm Gaussian FHWM algorithm in three dimensions. Our protocol stipulated that participants showing absolute or relative head motion exceeding 1 mm were excluded from further analyses, though no participants exceeded this threshold. In order to remove non-neuronal sources of coherent oscillation in the relevant frequency band (0.01–0.1 Hz), preprocessed data were subjected to probabilistic independent component analysis as implemented in MELODIC (Multivariate Exploratory Linear Decomposition into Independent Components) Version 3.10, part of FSL (FMRIB’s Software Library^1^). Noise components corresponding to head motion, scanner noise, and cardiac/respiratory signals were identified by observing their localization, time series, and spectral properties (as per Kelly et al., 2010) and removed using FSL’s regfilt command. Each participants’ functional data were coregistered to standard space (MNI 152 template) via registration of an averaged functional image to the high-resolution T1-weighted volume using a six degree-of-freedom linear registration and of the high-resolution T1-weighted volume to the MNI 152 template via non-linear registration, implemented in FNIRT.

### Designation of Regions of Interest

All networks were created by pooling from a set of 198 5 mm spherical ROIs; 196 of the ROIs were derived from a functionally derived cortical atlas (Power et al., 2011). We also included an additional pair of 5 mm ROIs centered on left (*x* = −22 mm, *y* = −6 mm, *z* = −14 mm) and right (*x* = 22 mm, *y* = −6 mm, *z* = −14 mm) amygdala, as this region was not included in the original cortical atlas. We used ROIs from the following networks defined by Power et al. (2011): visual (31 ROIs), fronto-parietal (25 ROIs), somatosensory motor (25 ROIs), dorsal attention (11 ROIs), ventral attention (nine ROIs), salience (18 ROIs), memory retrieval (five ROIs), cingulo-opercular (14 ROIs), and default mode (58 ROIs) Networks. ROIs were defined in MNI_152 standard space.

**FIGURE 1:**
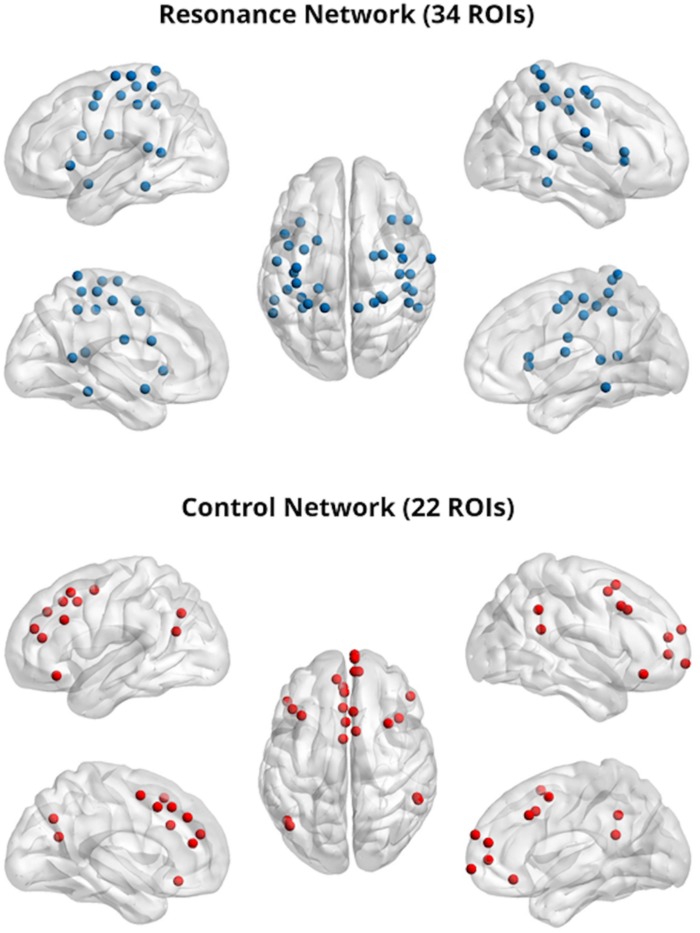
Resonance **(top)** and control **(bottom)** networks; 5 mm regions of interest were visualized with the BrainNet Viewer (http://www.nitrc.org/projects/bnv/) (Xia et al., 2013).

Two theory-driven networks (bottom-up resonance and top-down control) were also created by selecting ROIS from the Power cortical atlas overlapping with brain areas associated with neural resonance and top-down control. Resonance areas included the core cortical imitation circuitry (inferior frontal gyrus, inferior parietal lobule, superior temporal sulcus), as well as insular, limbic (bilateral amygdala), and somatomotor areas associated with neural resonance for visceral sensation, emotion, pain, and motor behavior (e.g. reviewed in Lamm et al., 2011; Zaki and Ochsner, 2012). This putative bottom-up resonance network consisted of 34 ROIs. Control areas included dorsolateral prefrontal cortex, TPJ, lateral orbitofrontal cortex, and sites covering a range from dorsal to ventral medial prefrontal and paracingulate cortex, implicated in top-down regulation of spontaneous and deliberate imitation, affect, and pain (Miller and Cohen, 2001; Banks et al., 2007; Decety and Lamm, 2007; Cho and Strafella, 2009; Spengler et al., 2010; Brighina et al., 2011; Volman et al., 2011; Tassy et al., 2012; Winecoff et al., 2013; Christov-Moore and Iacoboni, 2016; Christov-Moore et al., 2017b). This putative top-down control network consisted of 22 ROIs. This allowed us to test our conceptual model of resonance-control interaction as a substrate for empathic concern (Christov-Moore and Iacoboni, 2016; Christov-Moore et al., 2017b), while constraining ROI locations to those defined in Power et al. (2011) and assigning these ROI locations to the two networks on the basis of existing literature (see Table 1 for a list of ROIs used to define the resonance and control networks and Figure 1 for a visual rendering of the same ROIs/networks).

**TABLE 1:** MNI coordinates of powers cortical atlas ROIs employed in resonance and control networks.

**Region**	**Atlas ID**	**Network**	**MNI coordinates**		
			***X***	***Y***	***Z***
Inferior frontal gyrus pars opercularis	207	Resonance	48	22	10
	176	Resonance	−47	11	23
Anterior insula	208	Resonance	−35	20	0
	209	Resonance	36	22	3
Primary motor cortex	36	Resonance	42	−20	55
	29	Resonance	44	−8	57
	24	Resonance	−40	−19	54
	37	Resonance	−38	−15	69
Primary somatosensory cortex	27	Resonance	−38	−27	69
	26	Resonance	50	−20	42
	46	Resonance	66	−8	25
	45	Resonance	−53	−10	24
Inferior parietal lobule	33	Resonance	−45	−32	47
	190	Resonance	49	−42	45
	255	Resonance	47	−30	49
	259	Resonance	−33	−46	47
Superior parietal lobule	30	Resonance	−29	−43	61
	25	Resonance	29	−39	59
	22	Resonance	10	−46	73
	32	Resonance	22	−42	69
	38	Resonance	−16	−46	73
	34	Resonance	−21	−31	61
Premotor cortex	261	Resonance	−32	−1	54
	205	Resonance	42	0	47
	264	Resonance	29	−5	54
	174	Resonance	−44	2	46
Parahippocampal gyrus	125	Resonance	27	−37	−13
	126	Resonance	−34	−38	−16
Amygdala	N/A	Resonance	−22	−6	−14
	N/A	Resonance	22	−6	−14
Superior temporal sulcus	236	Resonance	−56	−50	10
	238	Resonance	52	−33	8
	240	Resonance	56	−46	11
	237	Resonance	−55	−40	14
Medial prefrontal/cingulate cortex	54	Control	7	8	51
	47	Control	−3	2	53
	213	Control	−1	15	44
	202	Control	−3	26	44
	112	Control	−2	38	36
	115	Control	−8	48	23
	113	Control	−3	42	16
	75	Control	6	67	−4
	216	Control	5	23	37
	105	Control	6	54	16
	106	Control	6	64	22
	108	Control	9	54	3
Dorsolateral prefrontal cortex	100	Control	−35	20	51
	193	Control	32	14	56
	196	Control	40	18	40
	201	Control	−42	25	30
Temporoparietal junction	79	Control	−46	−61	21
	204	Control	55	−45	37
	86	Control	−44	−65	35
	235	Control	54	−43	22
Orbitofrontal cortex	139	Control	49	35	−12
	137	Control	−46	31	−13

*IFGpo = inferior frontal gyrus pars opercularis.*

### Machine Learning Analyses

Mean BOLD time-courses were extracted from the average activity across voxels within each ROI. Matrices of pairwise Pearson correlation coefficients (operationalized here as connectivity weights) were created for each participant by correlating each ROI’s mean BOLD time-course with that of every other ROI within each network. Each of the non-redundant functional connectivity weights within the pairwise correlation matrices were concatenated into a single vector, creating a “feature set” for each participant. As such, each participant’s feature set consisted of $\frac{n\,\left(n-1\right)}{2}$ features, with *n* being the number of ROIs in the network(s) of interest, *n*-1 because diagonal identity correlations are not needed, and divided by two because the upper and lower parts of the matrices are symmetric. For “between-networks” analyses, ROIs belonging to each pair of networks being studied (e.g. Resonance and Control) were pooled in order to analyze the aggregate sets of ROIs as if they composed a single network, allowing for pairwise connectivity across all member ROIs of both networks.

To account for potential covariation, participant sex was iteratively regressed out of each feature and the residuals were subsequently used as the functional connectivity features. We implemented a leave-ten-subjects-out cross validation to assess the predictive power of network-specific feature sets. Specifically, we leveraged a least absolute shrinkage and selection operator (LASSO) regression model built on N-10 participants’ feature sets for each IRI subscale. The model’s intercept term and outcome beta values were then used as coefficients for each left-out subject’s feature set—obtaining a predicted subscale measure for that individual. After *N* folds, whereby each set of 10 participants was left out exactly once, we correlated the array of predicted values ($\hat{Y})$ with the actual values (*Y*), yielding Pearson’s *R*—a measure of our model’s feature-dependent ability to capture the behavioral variance across participants. We repeated this cross-validation 10 times and averaged the *R* values to converge on a true estimate of our test statistic, independent of which participants were randomly included in each fold. The LASSO regularization parameter was optimized before the leave-ten-subject-out cross-validation by using the least angle regression (LARS) algorithm on an *N* - 1 cross-validation that maximized the Pearson correlation between predicted values ($\hat{Y})$ with the actual values (*Y*) (Reggente et al., 2018).

### Significance Testing and Multiple Comparisons Correction

*R*-values from the *N*-10 cross-validation, averaged across the 10 iterations, were submitted to a significance test of the correlation coefficient ($t=\frac{r}{\sqrt{\frac{1-r^{2}}{N-2}}}$). In order to correct for multiple comparisons, we applied three family-wise corrections, for each set of hypotheses: (I) our main, theory-driven hypothesis that Resonance and Control interconnectivity predict Empathic Concern) and (II) our exploratory, broad hypothesis that trait empathy can be predicted from resting intra- and inter-connectivity of canonical and theory-driven intrinsic networks. Matrices of *p*-values within each family were created using a Benjamini–Hochberg approach (Benjamini and Hochberg, 1995) in *R* (*p*.adjust; method = “BH”; R Core Team, 2013) and corrected *p*-values were considered significant at the 5% positive-tail (i.e. *p* < 0.05). (Negative *R* values, indicating poor prediction accuracy—i.e. predicting a negative subscale score when the actual value is positive—are not readily interpretable).

### Data and Code Availability Statement

All data are freely available upon request. For human fMRI and behavioral data contact LC-M (leonardo.christovmoore@usc.edu). Custom scripts used in this analysis can be found at https://github.com/mobiuscydonia/Moore-2019-Empathic-Concern. Data and code sharing adopted by the authors complies with the requirements of our funding body as well as institutional ethics.

## Results

### Trait Empathy IRI Scores

We used a one-way ANOVA to examine whether the male and female participants differed significantly in self-reported trait empathy. Males and females did not differ significantly in Fantasizing (*F* = 2.68, *p* = 0.108), Empathic Concern (*F* = 2.59, *p* = 0.114), or Perspective-Taking (*F* = 0.274, *p* = 0.603). However, female subjects scored significantly higher on Personal Distress (*F* = 9.79, *p* = 0.003) (see Table 2).

**TABLE 2:** Means (with 95% confidence intervals) and standard deviations for each IRI subscale by gender.

	Male		Female	
	x̄ (95%CI)	σx̅	x̄(95%CI)	σx̅
FS	18.58 (16.54,20.61)	5.02	20.84 (18.89,22.79)	4.82
EC	22.61 (20.88,24.36)	4.31	24.54 (22.79,26.29)	4.34
PT	20.00 (17.88,22.12)	5.24	20.73 (18.78,2268)	4.82
PD	11.73 (9.41,14.05)	5.75	16.54 (14.39,18.69)	5.32

*Females scored higher on personal distress (PD), but not on PT (perspective-taking), FS (fantasizing) or EC (empathic concern).*

### Machine Learning and Connectivity

As described above, for these analyses, we examined 5 mm spherical regions of interest set in MNI_152 space for the visual, fronto-parietal, cingulo-opercular, dorsal and ventral attention, salience, memory retrieval, subcortical, somatomotor, and default mode networks (derived from Power et al., 2011) as well as two theory-driven networks (Resonance and Control, see Figure 1) created based on (a) a model of resonance-control interactions as a substrate for empathic concern (Christov-Moore and Iacoboni, 2016) and with ROIs derived from the literature as described above.

#### Within-Network Resting Connectivity Predicts Trait Empathy

**FIGURE 2:**
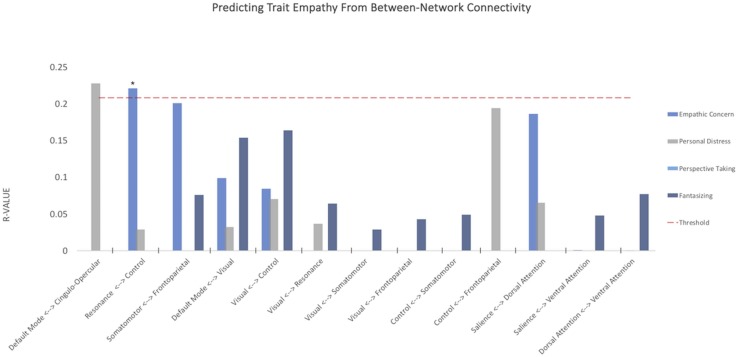
Within-network somatomotor resting connectivity predicts empathic concern. *Y*-axis depicts average correlations between values predicted from model trained on *n*-10 cross-validation set and remaining 10 subjects over multiple iterations. Red dashed line indicates threshold for *p* < 0.05, uncorrected. *p-value < 0.05 FDR corrected.

When examining the predictive power of connectivity weights *within* the selected intrinsic networks (Figure 2), empathic concern was significantly predicted by the somatomotor network (*R* = 0.374, *p* = 0.022, Benjamini–Hochberg false discovery rate (FDR) corrected). Personal distress was predicted above threshold by resonance (*R* = 0.236, *p* = 0.037, uncorrected), control (*R* = 0.22, *p* = 0.048, uncorrected), and cingulo-opercular networks (*R* = 0.242, *p* = 0.033, uncorrected); however, these did not survive FDR correction for multiple comparisons. None of the remaining subdimensions of empathy were significantly predicted by any of the within-network connectivity weights.

#### Between-Network Resting Connectivity Predicts Trait Empathy

When analyzing the predictive power of connectivity within and across multiple networks simultaneously, empathic concern was predicted by the between-network connectivity between the *a priori* resonance and control networks (*R* = 0.221, *p* = 0.0475, Benjamini–Hochberg FDR corrected), supporting the primary hypothesis of this study.

**FIGURE 3:**
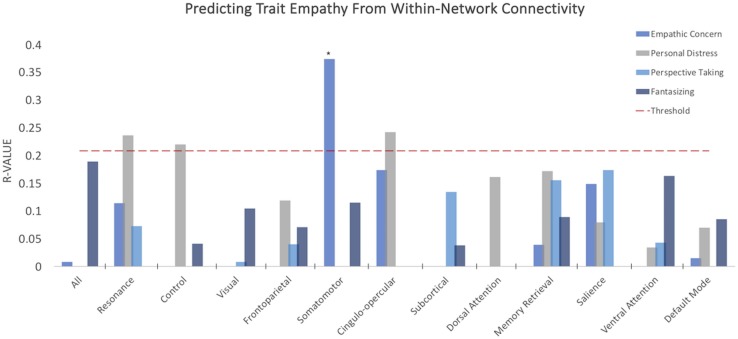
Between-network resting connectivity of resonance and control networks predicts empathic concern. *Y*-axis depicts average correlations between values predicted from model trained on *n*-10 cross-validation set and remaining 10 subjects over multiple iterations. Red dashed line indicates threshold for *p* < 0.05, uncorrected. *p-value < 0.05 FDR corrected.

When testing our second family of hypotheses, we examined whether subdimensions of empathic function could be predicted by connectivity within and across three types of network complexes: bottom-up resonance (visual/somatomotor, visual/frontoparietal, somatomotor/frontoparietal), resonance and control (control/frontoparietal, control/visual, control/somatomotor, cingulo-opercular/default mode), and links of no *a priori* interest as a comparison (dorsal/ventral attention, salience/dorsal attention), selected to test whether any of the subdimensions of empathic function could be predicted by differences in attentional networks (Figure 3). None of these survived FDR correction for multiple comparisons.

#### Control Analyses

To ascertain if these findings were indeed specific to functionally defined networks, we set out to create random, “sham networks,” where membership was not based on functional cohesion previously observed in the literature. As such, a random sampling without replacement from the pool of the 198 5 mm spherical ROIs across the whole brain was conducted to create two networks with equal numbers of ROIs as those in the networks that were significant and survived correction (i.e. 35 ROIs for the Somatomotor and Empathic Concern finding and 56 ROIs for the Resonance/Control and Empathic Concern result). These sham networks were submitted to the same iterative cross-validation procedure as our main analyses. We found that neither the sham somatomotor network (*r* = 0.018; *p* = 0.89) nor the sham resonance/control network (*r* = 0.004; *p* = 0.97) had significant power in predicting Empathic Concern.

## Discussion

In this study, we tested two hypotheses:

(I) We hypothesized that participants’ empathic concern for others would be predicted by resting connectivity between our theory-driven and literature-derived resonance and control networks.
(II) We hypothesized that we could predict subcomponents of participants’ trait empathy from the within- and between-network resting connectivity of canonical resting state networks.

As hypothesized in (I), participants’ levels of empathic concern were predicted by patterns of connectivity within and across the resonance and control networks (when treated as a single network), supporting the hypothesis (put forth in Christov-Moore and Iacoboni, 2016 and supported by Christov-Moore et al., 2017a) that these systems (a) continuously interact in a characteristic fashion observable in the absence of pertinent task demands and (b) this interaction is a likely neural substrate of empathic concern for others. Our findings (along with the previous work that prompted this study) support a dynamic, integrated view of empathic function, based on complex patterns of interaction between resonance and control systems rather than simply a univariate measure of overall connectivity. Indeed, numerous studies have reported task-related changes in connectivity between resonance and control networks during passive observation of emotions or pain (Christov-Moore and Iacoboni, 2016), reciprocal imitation (Sperduti et al., 2014), tests of empathic accuracy (Zaki et al., 2009), and comprehension of others’ emotions (Spunt and Lieberman, 2013). Interestingly, Raz et al. (2014) found evidence for complex, context-dependent interactions between “simulation” and “theory-of-mind” networks (largely corresponding to what are defined here as resonance and control networks) during empathic experience (observing films depicting personal loss). This multivariate approach may help reconcile findings supporting an integrated view with activation (e.g. Van Overwalle and Baetens, 2009) or lesion studies that suggest dissociated systems (e.g. Shamay-Tsoory et al., 2009): Lesions (transient/induced or physical) may simply be altering a crucial node for a specific integrated network outcome, just as a hand injury may affect the ability to catch a ball more than a back injury, though catching-like activities typically rely on hands, arms, and the core operating in unison. Indeed, the complexity of these interactions may be an obstacle to their efficient detection by standard activation or univariate connectivity methods. By employing flexible machine learning methods that make few *a priori* assumptions about the patterns of intrinsic connectivity underlying individual differences, we may achieve a more comprehensive multivariate view of the possible network-level patterns of neural interaction that give rise to individual differences in empathic function. It is common within cognitive neuroscience to theorize first about psychological processes and then investigate the neural correlates of such processes. However, in an exceedingly complex system such as the brain, much could be gained by approaching the problem from the opposite direction, by investigating how psychological processes *emerge* from brain organization (Fox and Friston, 2012).

As for (II), empathic concern was predicted by the within-network connectivity of the somatomotor network. This result further supports an embodied, somatomotor foundation for our concern for others’ welfare, in line with numerous findings relating vicarious somatosensory activation to multiple forms of prosocial behavior (non-strategic generosity in economic games: Christov-Moore and Iacoboni, 2016; harm aversion in moral dilemmas: Christov-Moore et al., 2017b; donations to reduce pain in another: Gallo et al., 2018; helping behavior: Hein et al., 2011; Masten et al., 2011; charitable donations: Ma et al., 2011). This also agrees with our recent finding that inferior premotor activation during observation of pain in others was predictive of participants’ later tendency to avoid inflicting harm in hypothetical moral dilemmas (Christov-Moore et al., 2017b). A major proposed subcomponent of empathy is fantasizing (Davis, 1983; Clay and Iacoboni, 2011), our ability to take the perspective of absent or fictional characters and become correspondingly invested in their welfare. Perhaps we implicitly construct internal models of others (present or implied/hypothetical) using perceptual, affective, and motor experiences we associate with past experience, framed by others’ intentions, moral character, group affiliation, etc. This embodied model of the “other” and its contextual framing would likely be represented by interactions between resonance and control processes, thus shaping the relative utility of their welfare (Bechara and Damasio, 2005), and hence the positive and negative reward values assigned to the outcomes of decisions that can affect them (Fehr and Camerer, 2007).

A clinical avenue suggested by this study is the potential ability to predict empathic functioning in populations that might have difficulty performing empathy tasks or filling out questionnaires, either due to being less cooperative or less cognitively able, e.g. in populations such as those with schizophrenia, low functioning autism, intellectual disabilities, or traumatic brain injury. Individuals in these groups might have, in principle, intact inherent capability for normal-range empathy that could be impeded by other limitations such as verbal or non-verbal communication (autism) or disorganized thought processes (schizophrenia); thus it would help us know what reasonable outcomes in terms of social and interpersonal functioning could be expected to result from therapies that help with training to rehabilitate or improve empathy, ultimately in the interest of enhancing social competence and social cognition. Indeed, it may be pertinent to include measures of empathic function along with standardized, multisite resting state scan protocols (like the Human Connectome Project), paving the way for a massive data-driven approach to produce models that can predict empathic function from the resting brain in many different populations.

### Limitations

While we have focused primarily on the patterns of functionally defined network activity underlying empathic concern, future work could make a similar theory-driven test of putative networks underlying other facets of empathic function (such as perspective-taking—a pursuit that did not succeed in this current work). Additionally, while we have shown that network properties, i.e. the aggregate of connectivity weights, can be used meaningfully as features to predict trait empathy, the nature of this multivariate approach does not readily provide simple conclusions about *what aspects of these networks* are predictive and in which direction. We cannot, for example, say: “increased interconnectivity predicts personal distress.” Graph theoretical analyses may allow for complementary mechanistic insights into the properties of whole networks, and parts of networks, that can predict trait empathy. Also, this study only examines “standard” connectivity, i.e. BOLD time-series correlation. Effective connectivity or mutual information analyses may shed light on more complex or non-linear interactions that might underlie the more dynamic, cognitive aspects of empathy (such as mentalizing or perspective-taking). Further, future larger studies could employ a whole-brain search that could potentially more broadly identify additional systems that contribute to empathy outside of the chosen networks implicated from previous studies.

## Conclusion

In conclusion, these findings support a dynamic, integrated model of the neural substrates for empathic concern. The presence of informative patterns of connectivity at rest suggests that these networks interact in a characteristic function regardless of task demands. Along the same lines, albeit at a more fine-grained, local level, these data support an embodied view of empathic concern, in which somatomotor representations of others’ harm situates our feelings and decisions about their welfare.

More broadly, these results add an important piece to a growing body of work demonstrating links between resting and task-positive brain function (Smith et al., 2009), suggesting that the two may not be as cleanly separable as is often implicitly assumed. Perhaps a multivariate, theory-driven method like the one employed here, combined with large datasets, could be applied to predict many aspects of cognition and behavior from resting brain activity. Having metrics that are stable, relatively context-invariant, and predictive of behavior is of great importance for the future of psychiatric research. Along these lines, finding markers of empathic functioning that are visible at rest may be of great potential prognostic and therapeutic utility, and could shed light on mechanisms underlying both healthy and abnormal empathic functioning.

## Data Availability Statement

The datasets generated for this study are available on request to the corresponding author.

## Ethics Statement

The studies involving human participants were reviewed and approved by the University of California Institutional Review Board. The patients/participants provided their written informed consent to participate in this study.

## Author Contributions

LC-M conceived the study, collected data, conducted data analysis, and prepared the manuscript. NR and PD conducted data analysis and aided in the preparation of the manuscript. JF aided in the preparation of the manuscript. MI was the primary investigator and aided in the preparation of the manuscript.

## Conflict of Interest

The authors declare that the research was conducted in the absence of any commercial or financial relationships that could be construed as a potential conflict of interest.

## Footnotes

1 www.fmrib.ox.ac.uk/fsl
